# Respiratory response to finger clamping in dogs under general anesthesia: A descriptive pilot study

**DOI:** 10.3389/fvets.2022.843956

**Published:** 2022-07-19

**Authors:** Lepape Sylvain, Sredensek Jerneja, Portier Karine

**Affiliations:** ^1^University Lyon, VetAgro Sup (Veterinary Campus of Lyon), 69280, Marcy l'Etoile, France; ^2^University Lyon, CarMeN Laboratory, INSERM, INRA, INSA Lyon, Université Claude Bernard Lyon, Bron, France

**Keywords:** nociception, Drive, Timing, dog, anesthesia, ventilation

## Abstract

**Aim of the study:**

The aim of this study was to assess the effects of a nociceptive stimulus on respiratory variables in anesthetized dogs.

**Material and method:**

Eleven dogs received acepromazine administered intramuscularly (IM) at a dose of 0.04 mg kg^−1^ 45 mins before induction of anesthesia. Loss of consciousness was obtained with midazolam at 0.2 mg kg^−1^ and propofol administered at a dose of 2 mg kg^−1^ intravenously (IV). Orotracheal intubation was performed and anesthesia was maintained with isoflurane in 100% oxygen. Inspired (V_Ti_) and expired (V_Te_) tidal volume (V_T_), minute volume (V_M_), inspiratory and expiratory time (Ti; Te) were measured and recorded twice a second by a spirometer. The Drive (V_T_/T_i_) and Timing [Ti/(Ti+ Te)] were calculated.

After stabilizing the depth of anesthesia the variables measured by the spirometer were recorded for 5 mins [T_0−5_-T_0_]. Then (T_0_) interdigital clamping of the hind leg was performed until a withdrawal movement was observed. If no reaction occurred, the clamp was left in place for 60s. After removal of the clamp, respiratory variables were measured continuously for another 5 mins [T_0_-T_0+5_]. At T_0+5_ morphine (0.2 mg kg^−1^ IV) was administered. Five minutes later (T_0+10_), a second clamp test was performed, using the same procedure. At T_0+15_ the data recording was stopped.

**Result:**

The results showed a large variation in the individual values of Drive and Timing and are presented in a descriptive manner. The observation of Drive values over time showed variations following nociceptive stimuli. Drive appears to have increased only for those dogs that did not move during the stimulus, and were therefore pinched for a full 60 s. In contrast, the study of the Timing values revealed no difference between the data before and after nociceptive stimulation. However Timing seems to increase after morphine administration.

**Conclusion:**

Drive remains a parameter that needs to be studied in depth to determine its sensitivity and precocity to monitor acute nociception.

## Introduction

The nociceptive response to surgery is characterized by many widely described adverse effects in humans and animals (1). These effects may increase the incidence of postoperative morbidity and mortality (2). Therefore, during general anesthesia, it is essential to be able to rely on early and reliable indicators of nociception to minimize these adverse postoperative consequences.

There is currently still no proven method to objectively measure acute pain intensity. Several devices, already on the market, have been developed in humans and animals with the aim of objectifying the monitoring of the nociception/anti-nociception balance under general anesthesia. However, their scope is restricted to certain conditions and there is no clinical evidence that their use improves patient outcomes (3).

More traditionally, vital signs, including hemodynamic and respiratory variables have been studied in veterinary medicine as potential indicators of nociception (4, 5). However, in dogs, there is little evidence to show a linear and reliable correlation between these physiological factors and nociception. While an increase in systemic blood pressure seemed to indicate a nociceptive response (6), changes in heart rate or respiratory rate were not significantly associated with nociception (7).

In humans, variation in lung ventilation variables have been proposed as markers of intraoperative nociception. Eger et al. (8) observed a reduction in minute ventilation (V_M_) and tidal volume (V_T_) without any increase in respiratory rate during anesthesia under isoflurane while the surgical stimulus, under the same volatile agent, induced an increase in minute volume mainly due to an increase in respiratory rate. Sutherland and Drummond (9) recorded an increase in respiratory inspiratory flow due to a marked increase in V_T_ after skin incision. Dockerty and Drummond (11), showed that the respiratory response to a nociceptive stimulus consisted of an increase in mean inspiratory flow rate [respiratory DRIVE, an index defined as the ratio of tidal volume to inspiratory time (V_T_/Ti)]. They also showed an increase in fractional inspiratory time [respiratory TIMING, an index defined as the ratio of Ti to total (T_T_=Ti+Te) respiratory cycle time (Ti/T_T_)]. However, these effects have not been found in horses (12), and, to our knowledge, have never been studied in dogs.

The aim of this study was to assess the effects of a nociceptive stimulus on respiratory Drive (V_T_/Ti) and Timing (Ti/T_T_). We hypothesized that interdigital clamping will affect the Drive but not the Timing and that morphine administration will attenuate this effect.

## Materials and methods

This study was registered with the VetAgro Sup ethics committee n°18 (project N°1708). Informed consent was obtained from the owner before data collection began.

### Animals

Eligibility criteria, over a period of 3 months, were that the dog was presented to the anesthesia service for elective surgery (ovariectomy and castration), was in good health, was not brachycephalic and weighed between 10 and 20 kg. Dogs were judged healthy by clinical examination (with particular attention to the respiratory system and any pre-existing pain), as well as by normal values for haematocrit and total protein. Only dogs that were pain-free and classified as I in the American Society of Anesthesiologists (ASA) physical status classification, were included in the study. Animals requiring additional sedation to the standard protocol were excluded from the study.

**Figure 1:**
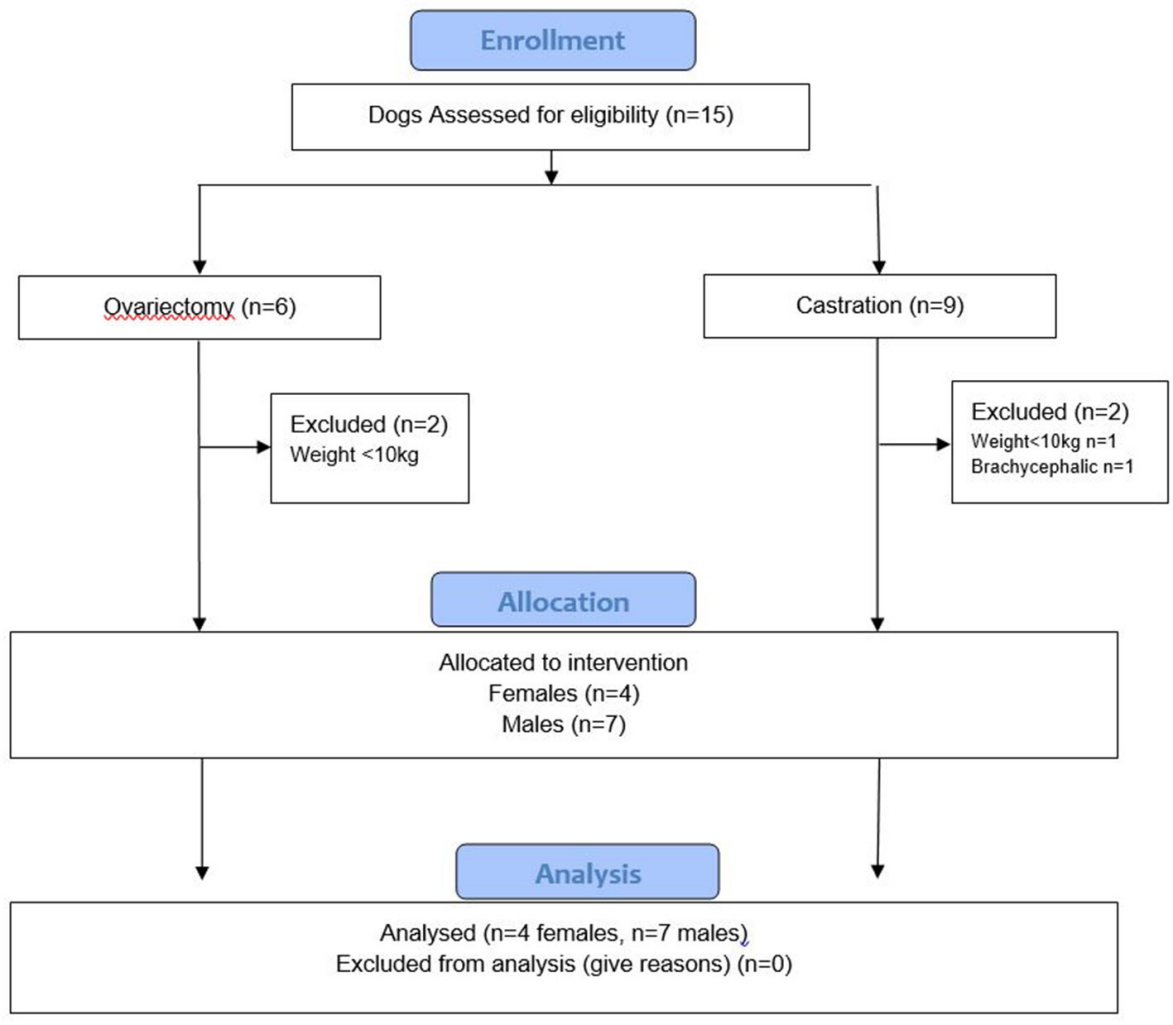
Flow diagram. Cases recruitment.

Fifteen dogs (6 undergoing ovariectomy and 9 undergoing castration) were assessed for eligibility and 11 (four females and seven males, on average median [min; max] 1 year and 10 month old [6 months; 7 years and 10 months], weighing on average median [min; max] 17 kg [6.5; 24.9]) were finally included in the analysis (Figure 1).

### Anesthesia

The dogs were fasted for 12 h with free access to water until 2 h before surgery. Left or right cephalic vein was catheterized.

Acepromazine (Calmivet, Vetoquinol, Paris, France) was administered intramuscularly (IM) at a dose of 0.04 mg kg^−1^ 45 mins before induction of anesthesia. Loss of consciousness was obtained with midazolam (Midazolam, Mylan, Saint Priest, France) at 0.2 mg kg^−1^ given slowly intravenously (IV) and propofol (PropoVet Multidose, Zoetis Axience, Paris, France), administered at a dose of 2 mg kg^−1^ IV and then titrated to effect with extra boli of 0.5 mg kg^−1^ if necessary. Orotracheal intubation was performed and anesthesia was maintained with isoflurane (Isoflurin, Vetpharma Animal Health S.L., Barcelona, Spain) delivered *via* a calibrated vaporizer (Isotec 5, Datex-Ohmeda, Limonest, France) set at 2% in 100% oxygen (flow rate 1L minute^−1^), an anesthetic machine (Moduflex, Dispomed, Dinan, France) and a small animal rebreathing circle circuit (en Y, Dispomed, Dinan, France). Dogs were positioned in right lateral recumbency. The depth of anesthesia was considered adequate in the absence of movement and when the eyes were tilted ventromedially and the jaw tone had disappeared. If necessary, a bolus of 0.5 mg kg^−1^ of propofol was administered and the number of extra boli of propofol was recorded. The anesthetics were performed and recorded by the same anesthetist for all dogs.

### Monitoring

Variables such as heart rate (HR), respiratory frequency (f_R_), peripheral oxygen saturation of hemoglobin (SpO_2_), systolic, diastolic and mean non-invasive arterial blood pressure (SAP, DAP and MAP, respectively) measured by oscillometry and body core temperature (T°C), measured by an esophageal probe, were monitored by a pre-calibrated multi-parametric monitor (PVM-2703, Nihon Kohden, Rosbach, Germany) and recorded every 5 mins on the anesthetic record.

Inspired (V_Ti_) and expired (V_Te_) tidal volume (V_T_), minute volume (V_M_), inspiratory and expiratory time (Ti; Te) were measured and recorded twice a second by a spirometer (Citrex, Rigel Medical, Seaward Group,Tampa, USA) placed between the endotracheal tube and the anesthesia circuit.

The inspiratory gas flow rate i.e., Drive [(V_Ti_+V_Te_)/2)/Ti in ml s^−1^) and the ratio between Ti and total respiratory cycle time (T_T_) i.e., Timing [(Ti/(Ti +T_e_)] without unit] were calculated for each measurement taken by the spirometer.

Given the variability of the Drive values among the dogs, the Drive was first related to the weight of each dog, in order to obtain a Drive value in ml s^−1^ kg^−1^. The Drive was then related to its variation from its initial mean value, by dividing the “Drive” value at time t by the mean “Drive” calculated over the period of interest ([1–4 mins 30 s] before first clamping;[5 mins 56 s−9 mins 26 s] before morphine;[10 mins 56 s−14 mins 26 s] before second clamping). We thus obtained values of variation of the Drive with respect to the average at each time t of the measurement.

### Study design

After stabilizing the depth of anesthesia, heart rate and respiratory rate, the variables measured by the spirometer were recorded for 5 mins [T_0−5_-T_0_]. Then (T_0_) a hemostatic clamp was clamped (to the first notch) between two fingers of the left hind leg until a withdrawal movement was observed. If no reaction occurred, the clamp was left in place for 60s. After removal of the clamp, respiratory variables were measured continuously for another 5 mins [T_0_-T_0+5_]. At T_0+5_ morphine (Morphine Clorhydrate Aguettant, Aguettant Laboratory, France) 0.2 mg kg^−1^ IV was administered. Five mins later (T_0+10_), a second clamp test was performed, using the same procedure. At T_0+15_ the data recording was stopped.

Anesthesia was then continued and analgesia was provided as required to allow the surgery to be performed.

### Statistics

The data were analyzed with R software (10). R: A language and environment for statistical computing. R Foundation for Statistical Computing, Vienna, Austria).

Observation of the results showed a large variation in individual Drive and Timing values in response to the stimulus over time. For these reasons, we chose to present the results in a descriptive manner.

### Power calculation

Dockerty and Drummond (11) showed a 17% increase in tidal volume in man after surgical incision. This corresponds to an increase of about 2 ml kg^−1^ in dogs if we consider that the average tidal volume in dogs is 12.06 ml ± 1.11 ml kg^−1^ (13). Sample size calculation (considering that the standard deviation of tidal volume in the dog population is 1.1 ml kg^−1^) indicated that a total of 9 dogs were required if the true difference in tidal volume is 2 ml kg^−1^ after clamping, with 0.95 power and 0.05 alpha level.

## Results

Of the 11 dogs, six did not show any paw withdrawal movement during the first clamping.

**Figure 2:**
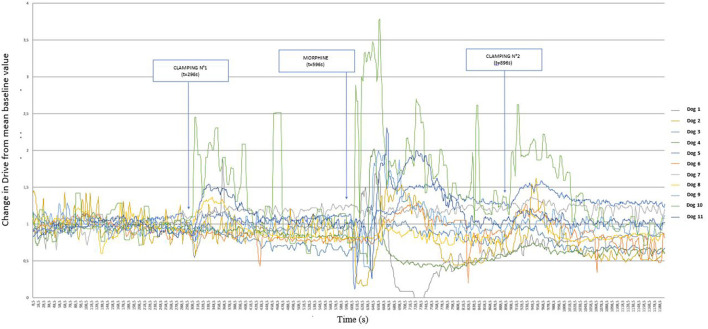
Graphical representation of the variation of Drive [((V_Ti_+V_Te_)/2)/Ti/weight of the dog] (ml s^−1^ kg^−1^) from its baseline mean value as a function of time in 11 healthy dogs placed under general anesthesia and undergoing two interdigital space clamp stimuli separated by morphine injection (0.2 mg kg^−1^). Inspired (V_Ti_) and expired (V_Te_) tidal volume, inspiratory and expiratory time (Ti; Te).

During the first clamp, the reading of Figure 2 suggests that a number of dogs had increased Drive values (since the values of variations of this parameter are >1), with different intensities.

**Figure 3:**
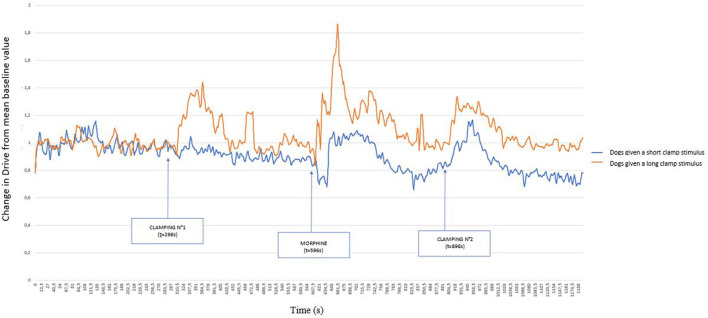
Graphical representation of the variation of the Drive [((V_Ti_+V_Te_)/2)/Ti/weight of the dog] (ml s^−1^ kg^−1^) from its baseline mean value as a function of time in 11 healthy dogs placed under general anesthesia and undergoing two clamping stimuli of the interdigital space separated by morphine (0.2 mg kg^−1^) injection. The dogs were divided into two groups: those that withdrew their paw at the time of the first clamping (short stimulus) and those for which the clamp remained in place for 1 min (long stimulus). Inspired (V_Ti_) and expired (V_Te_) tidal volume, inspiratory and expiratory time (Ti; Te).

Figure 3 represents these same variations, but distinguishing between dogs that showed a paw withdrawal movement (dog 1, 5, 8, 9 10, 11) and those that did not move (dog 2, 3, 4, 6,7), undergoing the clamp for 60 s. From the comparison of these two graphs, the early removal of the paw prevented the variation of the Drive, whereas the clamping for 60 s caused a marked variation of the Drive.

**Figure 4:**
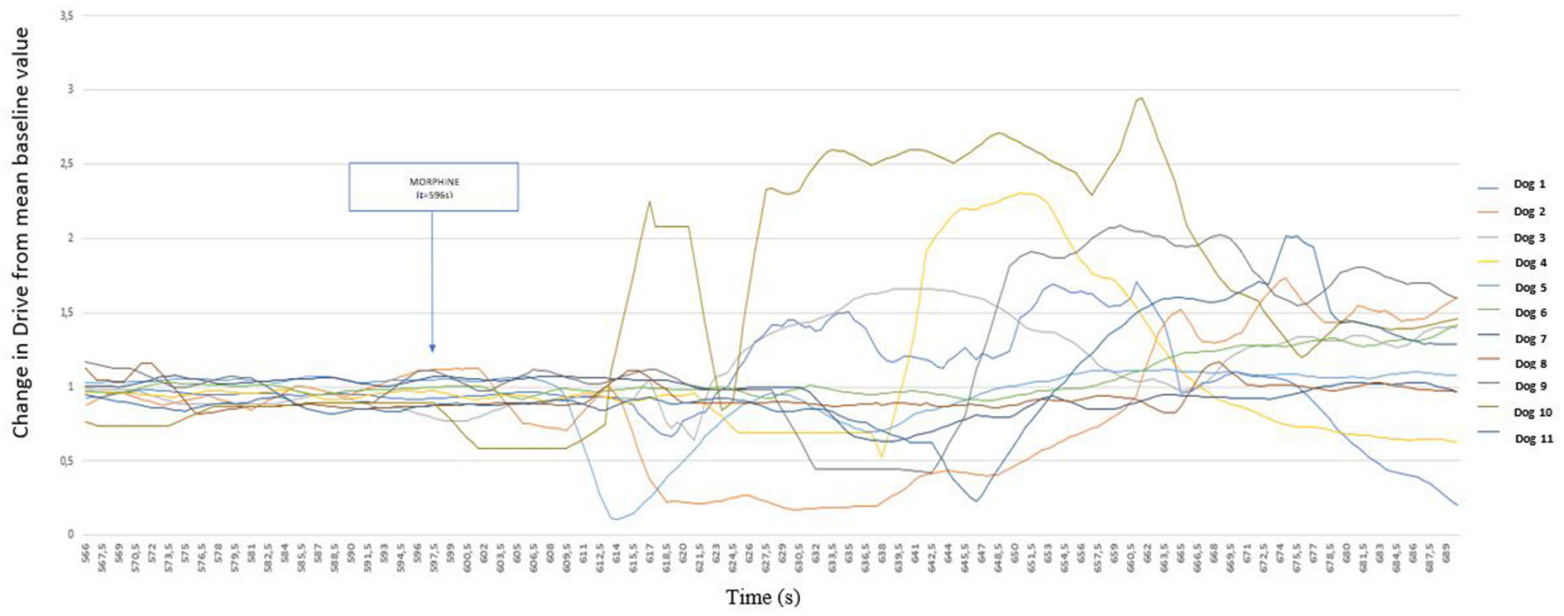
Detailed representation of the variation of Drive [((V_Ti_+V_Te_)/2)/Ti/weight of the dog] (ml s^−1^ kg^−1^) from its baseline mean value as a function of time before and after morphine injection (0.2 mg kg^−1^) in 11 healthy dogs placed under general anesthesia Inspired (V_Ti_) and expired (V_Te_) tidal volume, inspiratory and expiratory time (Ti; Te).

In response to the morphine injection, the Drive varied in a biphasic manner within 1 min of injection, decreasing first and increasing thereafter. Figure 4 details the period following the injection.

**Figure 5:**
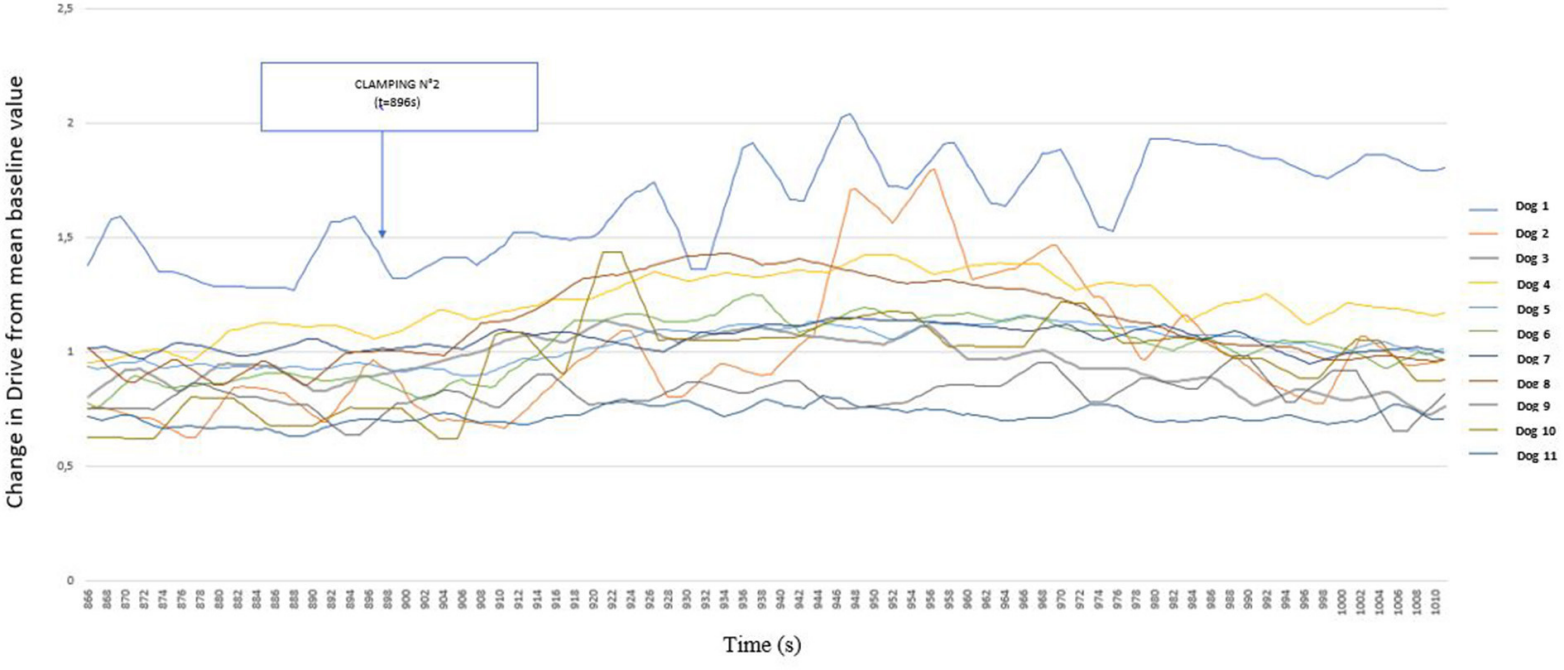
Detailed representation of the variation of Drive [((V_Ti_+V_Te_)/2)/Ti/weight of the dog] (ml s^−1^ kg^−1^) from its baseline mean value as a function of time before and after clamping of the interdigital space in 11 healthy dogs placed under general anesthesia after morphine administration (0.2 mg kg^−1^). Inspired (V_Ti_) and expired (V_Te_) tidal volume, inspiratory and expiratory time (Ti; Te).

Figure 5 shows that Drive values increased in nine dogs following the second nociceptive stimulation. It is also interesting to note that during the second clamping episode, no dog showed a withdrawal reflex, and all nociceptive stimuli lasted 60 s for this second manipulation.

**Figure 6:**
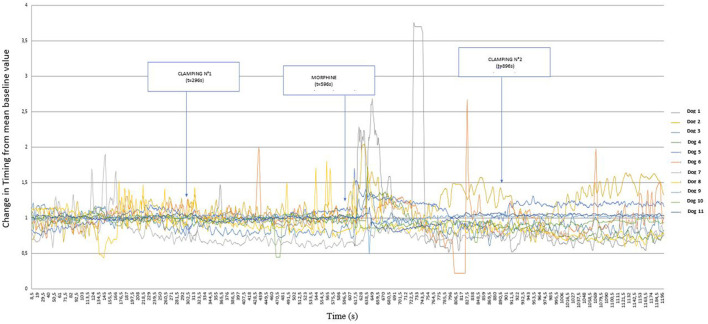
Graphical representation of the variation of Timing [Ti/(Ti +T_e_)] from its baseline mean value as a function of time in 11 healthy dogs placed under general anesthesia and undergoing two interdigital space clamp stimuli separated by morphine injection (0.2 mg kg^−1^). Inspired (V_Ti_) and expired (V_Te_) tidal volume, inspiratory and expiratory time (Ti; Te).

The variations in Timing with respect to the stimuli are shown in Figure 6. The different stimuli appear to produce smaller, and more variable, reactions depending on the dog. However this figure shows an increase in Timing after morphine administration.

## Discussion

Although no differences could be shown, the observation of Drive values over time suggested variations following nociceptive stimuli. The study of the effect of clamping and morphine administration on Timing values revealed smaller and more variable results than the Drive values.

Interestingly, the type of response to clamping had different effects on the evolution of Drive values. Visual analysis shows that the Drive increased only for those dogs that did not move during the stimulus, and were therefore pinched for a full 60 s. The withdrawal reflex is a so-called “nociceptive” reflex, which involves nociceptive receptors. Bergadano et al. (14) suggested that it may allow the quantification of the excitability of the nociceptive system and the efficacy of analgesics in conscious dogs. The lack of an increase in Drive in dogs that have shown a paw withdrawal reflex is therefore likely to be a result of a nociceptive stimulus that is too short, or of too low in amplitude. It is possible that in the dogs that withdrawn their paw, only the A-delta fibers were stimulated as the clamp was removed early. Whereas the persistence of the clamp for 1 min may have resulted in stronger nociceptive stimulation, inducing activation of the C-fibers which caused nociception that was sufficiently intense and diffuse to trigger a ventilatory response (15).

Likewise, the type of stimulus used in our study may not have been appropriate to induce a change in respiratory variables in all dogs.

To produce a test nociceptive stimulus, the technique of pinching the space between the toes on paws or applying a pressure over a finger with a Halstead clamp or hemostat was widely used in past studies (16, 17). Nevertheless, it is known that a mechanical stimulus (such as pinching a skin area, or a finger) causes the activation of low-threshold mechanoreceptors, making this test non-specific (18).

On the other hand, electrical stimulation has the disadvantage of stimulating all peripheral nerve fibers, including fibers not involved in nociception, which handle other nerve impulses (14, 18). In 2003 Valverde (19) validated several types of noxious stimuli for use in determining the minimum alveolar concentration for inhalation anesthetics in dogs and rabbits. They concluded that clamping and electrical stimulation are both supramaximal stimuli and that there were no significant differences between techniques and sites of application.

Therefore both electrical and mechanical stimulations are comparable in terms of non-specificity and inability to reproduce an event encountered by an animal, but electrical stimulation offers the advantage of being reproducible, quantifiable and non-invasive.

A study (20) in deeply anesthetized rabbits revealed that low- and high-frequency tooth pulp stimulation evoked respiratory response only when it was associated with muscular contractions of the digastric muscle. A study, performed by Conde Ruiz et al. (12), in horses undergoing arthroscopy, showed no variation in Drive or Timing. The entry into the joint, at the fetlock level, did not involve muscular fibers or muscular contraction. The author concluded that this may explain, among other things, the lack of variation in Drive and Timing observed in their study. In view of these results, it might be interesting to reproduce our study and observe the evolution of Drive and Timing in response to the stimulation of a muscle as a nociceptive stimulus, for example during laparotomy.

Morphine injection appears to have induced a biphasic Drive response in most dogs, resulting in a decrease followed by an increase in Drive values. Morphine act on central receptors involved in the regulation of ventilation, particularly affecting tidal volume (21). When administered to conscious healthy dogs, morphine causes a significant dose-dependent increase in respiratory rate (as it causes the dog to pant) and minute ventilation, associated with a decrease in tidal volume (21). This decrease in tidal volume could explain the decrease in the drive observed in the first instance. However, the injection of morphine seems to have induced an increase in Timing which may be the consequence of an increase in inspiratory time or a decrease in expiratory time or the sum of both. We found no support in the literature for these assumptions about the effect of morphine on inspiratory or expiratory times.

### Limitations of the study

It appears that clamping 5 mins after morphine administration induced a slight increase in Drive values in most dogs. Morphine therefore did not appear to be sufficiently effective in inhibiting the response to clamping. There is evidence that morphine is detected in its conjugated form in plasma within 1.5 to 2.5 mins after intravenous administration and is detectable in CSF within 2–5 mins. However peak CSF concentration is observed between 15 and 30 min after injection (22). Maybe an additional waiting time before the second clamp might have allowed a better antinociceptive action of morphine.

The anesthetic molecules (acepromazine, midazolam, propofol and isoflurane) are unlikely to have affected the results as they do not have an analgesic effect. Nevertheless, their respiratory depressant effect may have affected the respiratory effect of the stimuli compared to what would have been observed in non-anesthetized dogs.

It would be necessary to include a larger number of dogs to confirm the trends observed on the curves. Our power calculation was based on the tidal volume and not directly on the Drive or Timing as this was the only value available in the literature in dogs.

In addition, it should be noted that we cannot exclude subclinical respiratory disease because the preoperative examination was not based on chest X-rays or further blood tests.

The dogs acted as their own control before and after the stimuli. It might have been interesting to form a control group to compare the effect of the second clamp without morphine. The latency and response times differ from one individual to another even if the curves are similar. This is why an individual descriptive approach seemed more interesting to us.

## Conclusion

The results of our study do not allow us to state with certainty whether the parameters Drive and Timing are good indicators of nociception or not. Drive remains a parameter that needs to be studied in depth to determine its sensitivity and precocity. Further studies need to be conducted to determine if Drive might be a valuable parameter to include in the development of advanced acute pain monitors.

## Data availability statement

The original contributions presented in the study are included in the article/supplementary material, further inquiries can be directed to the corresponding author/s.

## Ethics statement

The animal study was reviewed and approved by VetAgro Sup Ethic Committee. Written informed consent was obtained from the owners for the participation of their animals in this study.

## Author contributions

LS, SJ, and PK participated in conception of the work, data acquisition, interpretation, wrote or contributed to the writing of the manuscript, and revised the manuscript. LS and PK performed data analysis. All authors contributed to the article and approved the submitted version.

## Conflict of interest

The authors declare that the research was conducted in the absence of any commercial or financial relationships that could be construed as a potential conflict of interest.

## Publisher's note

All claims expressed in this article are solely those of the authors and do not necessarily represent those of their affiliated organizations, or those of the publisher, the editors and the reviewers. Any product that may be evaluated in this article, or claim that may be made by its manufacturer, is not guaranteed or endorsed by the publisher.
